# Development of Selectable Marker-Free Transgenic Rice Plants with Enhanced Seed Tocopherol Content through FLP/*FRT*-Mediated Spontaneous Auto-Excision

**DOI:** 10.1371/journal.pone.0132667

**Published:** 2015-07-14

**Authors:** Hee-Jong Woo, Yang Qin, Soo-Yun Park, Soon Ki Park, Yong-Gu Cho, Kong-Sik Shin, Myung-Ho Lim, Hyun-Suk Cho

**Affiliations:** 1 Biosafety Division, National Academy of Agricultural Science, Rural Development Administration (RDA), Jeonju, Republic of Korea; 2 School of Applied Biosciences, Kyungpook National University, Daegu, Republic of Korea; 3 Department of Crop Science, Chungbuk National University, Cheongju, Republic of Korea; National Institute of Plant Genome Research, INDIA

## Abstract

Development of marker-free transgenic plants is a technical alternative for avoiding concerns about the safety of selectable marker genes used in genetically modified (GM) crops. Here, we describe the construction of a spontaneous self-excision binary vector using an oxidative stress-inducible modified FLP/*FRT* system and its successful application to produce marker-free transgenic rice plants with enhanced seed tocopherol content. To generate selectable marker-free transgenic rice plants, we constructed a binary vector using the *hpt* selectable marker gene and the rice codon-optimized *FLP* (*mFLP*) gene under the control of an oxidative stress-inducible promoter between two *FRT* sites, along with multiple cloning sites for convenient cloning of genes of interest. Using this pCMF binary vector with the *NtTC* gene, marker-free T_1_ transgenic rice plants expressing *NtTC* were produced by *Agrobacterium*-mediated stable transformation using hygromycin as a selective agent, followed by segregation of selectable marker genes. Furthermore, α-, γ-, and total tocopherol levels were significantly increased in seeds of the marker-free transgenic TC line compared with those of wild-type plants. Thus, this spontaneous auto-excision system, incorporating an oxidative stress-inducible mFLP/*FRT* system to eliminate the selectable marker gene, can be easily adopted and used to efficiently generate marker-free transgenic rice plants. Moreover, nutritional enhancement of rice seeds through elevation of tocopherol content coupled with this marker-free strategy may improve human health and public acceptance of GM rice.

## Introduction

Rice is one of the most important food crops consumed worldwide. Rice is also a good source of vitamin E, an essential lipid-soluble nutrient that consists of four tocopherols and four tocotrienols. Each of these types of compounds has α-, β-, δ-, and γ-forms determined by the number of methyl groups on the chromanol ring. Tocopherols can efficiently quench singlet oxygen and scavenge various radicals, particularly lipid peroxy radicals, thereby terminating lipid peroxidation chain reactions [1, 2]. Tocopherols are important constituents of the human diet and have been shown to aid in immune function [3] and to decrease the risk of a number of degenerative diseases, such as Parkinson’s disease [4] and heart disease [5]. Additionally, recent reports have demonstrated that tocopherols can affect important physiological processes in plants, such as germination, photoassimilate export, growth, and leaf senescence; tocopherols also have antioxidant functions in photosynthetic membranes and play important roles in plant responses to abiotic stresses [6].

In addition to its importance as a source of vitamin E, rice is also an important model system for functional identification of genes in monocots. Several transformation systems have been developed in rice plants using different approaches, such as protoplast transformation, particle bombardment transformation, and *Agrobacterium*-mediated transformation methods [7–9]. The advantages of *Agrobacterium*-mediated gene transfer over other methods include the high efficiency of transformation, the ability to transfer relatively large segments of DNA, and the relatively short duration of transformation [10]. Thus, *Agrobacterium*-mediated methods have become popular in recent years.

In general, antibiotic- or herbicide-resistant genes linked to a transgene have been used as selectable marker genes for the preferential growth of transformed cells in transformation systems. However, once transformation is accomplished, the presence of these resistance genes is no longer necessary and can even be undesirable. Moreover, the presence of selectable marker genes in genetically modified (GM) crops has generated a number of environmental and consumer concerns, despite scientific evidence supporting the safety of GM crops [11]. However, regardless of scientific assurance of the safety of selectable marker genes, consumer acceptance is ultimately the most important element determining the successful commercialization of transgenic plants and products. Therefore, marker gene elimination is desirable in order to alleviate public concerns over the safety of food derived from transgenic crops.

Several strategies have been developed to generate selectable marker-free transgenic plants genes using different transformation systems. These strategies include co-transformation, site-specific recombination, transposon-based methods, and intrachromosomal homologous recombination [11–14]. The principle of the co-transformation strategy is the introduction of a selectable marker gene and a gene of interest from different T-DNAs. If the two genes are integrated into unlinked loci, subsequent segregation can separate the gene of interest from the selectable marker gene. Marker genes of transgenic plants can also be removed by site-specific recombination systems, such as Cre/*lox*, FLP/*FRT*, and R/*RS*. Several types of site-specific recombination systems function in plants [11]. Among these, the Cre/*lox* system from bacteriophage P1 has been most extensively used for the generation of marker-free plants. Moreover, strategies for generation of marker-free plants via site-specific recombination systems require either the transient expression of the recombinase gene, crossing with a recombinase-expressing line, or an inducible factor to turn on the expression of the recombinase gene. Among these methods, auto-excision using an inducible promoter has been developed due to the advantages of reduced time requirements of avoidance of crossing procedures. Several inducible systems responsive to external stimuli have been reported for plants. The heat-shock regulated system has been shown to be functional in *Arabidopsis* [15], tobacco [16], potato [17], maize [18], rice [19], and aspen [20]. The chemically regulated self-excision system, i.e., combination of the *Cre* gene with the XVE system, has been successfully applied in *Arabidopsis* [21], rice [22], and tomato [23, 24]. In another FLP/*FRT* system from *Saccharomyces cerevisiae*, the selectable marker gene of transgenic tobacco was removed by a stress-inducible FLP/*FRT* site-specific recombination system [25]. In this system, an oxidative stress-inducible peroxidase (POD) promoter is fused to the recombinase gene *FLP*. Hydrogen peroxide is then used to induce expression of the recombinase gene, and successful excision of selectable marker genes via hydrogen peroxide-regulated site-specific recombination is achieved. Despite the advantages of temporally regulated recombinase expression, heat-shock or chemically regulated promoters require an external signal to be activated, and the recombination frequencies are greatly dependent on the penetration of the signal into plant cells [26]. Therefore, these systems may be needed further improvements for broad applications in the agricultural industry.

In our present study, we describe the construction of a spontaneous self-excision binary vector using the oxidative-stress inducible *mFLP*/*FRT* system for rice transformation. In a recent report, Woo et al. [27] showed that overexpression of *NtTC* could increase the tocopherol contents in leaves of rice plants. Therefore, for efficient generation of marker-free transgenic rice plants via the spontaneous auto-excision method with enhancement of the tocopherol content of rice seeds, we generated marker-free T_1_ transgenic rice plants overexpressing *NtTC* by *Agrobacterium*-mediated transformation using hygromycin as a selective agent, followed by segregation of selectable marker genes and analysis of tocopherol content.

## Materials and Methods

### Vector construction and codon optimization

Molecular manipulation methods, such as plasmid DNA isolation, restriction enzyme analysis, ligation of DNA fragments, and transformation of *Escherichia coli*, were performed as described previously [28]. The binary vectors for this study were constructed as follows. The 6.6-kb backbone DNA fragment for binary vector construction was obtained by polymerase chain reaction (PCR) from the pCAMBIA2301 vector (CAMBIA, Canberra, Australia) with the following primers, incorporating the *Hin*dIII and *Sac*I sites: forward primer, 5′-AAGAGCTCAACGTCCGCAATGTGTTATTAAGTTGTCTAAGCG-3′ and reverse primer, 5′-AAAAGCTTGGTGACCAGCTCGAATTTCCCCGA-3′. The 1,817-bp DNA fragment containing the P35S-*FRTm*-*hpt*-T35S cassette and 2,964-bp Ppod-*FLP*-Tnos-MCS cassette were synthesized (Bio Basic Inc., Markham, Canada). The P35S-*FRTm*-*hpt*-T35S cassette was excised with *Sac*I and *Spe*I and inserted into the same restriction enzyme sites of the pBluescript II KS vector (Stratagene, La Jolla, CA). The Ppod-*FLP*-Tnos-MCS fragment was excised with *Spe*I and *Hin*dIII and subcloned into the *Spe*I/*Hin*dIII site of the pBluescript-P35S-*FRTm*-*hpt*-T35S plasmid to generate pBluescript-P35S-*FRTm*-*hpt*-T35S-Ppod-*FLP*-Tnos-MCS. The insertion fragment was then excised from this construct with *Sac*I and *Hin*dIII and ligated with the backbone fragment treated with *Sac*I/*Hin*dIII restriction enzymes to generate the binary vector pHWMF.

The FLP recombinase gene originated from a 2-μm plasmid in *S*. *cerevisiae*. To optimize codon usage, the relative synonymous codon usage (RSUC) values of *Oryza sativa* and the native *FLP* gene were calculated using the information obtained from the codon usage database (http://www.kazusa.or.jp/codon/). RSUC values were calculated by dividing the observed codon usage by that expected when all codons for the same amino acids were used equally [29]. The entire *mFLP* gene was synthesized based on the RSUC values of *O*. *sativa* and the codon usage of the rice high-GC gene [30]. The synthetic *mFLP* gene contained the *Bgl*II sites at the 5′ and 3′ ends to facilitate gene exchange. The *mFLP* gene, excised from the pUC57 vector with *Bgl*II, was inserted into the corresponding *FLP* gene site of the pHWMF vector. The resulting binary expression vector, designated as pCMF, was verified by DNA sequencing and restriction enzyme analysis. The full-length *NtTC* gene (Genbank accession number KJ645980) was obtained from pMJ102TC [27] by PCR with the following primers incorporating *Bam*HI and *Hin*dIII sites: forward primer, 5′-TTGGATCCATGGAGAACATATACGATTTTTCCACCATCTCTTC-3′ and reverse primer, 5′-TTAAGCTTACAGGCCAGGAGGTTTGAGAAATGGAG-3′. The gene was then inserted into MCS of the pCMF vector with *Bam*HI and *Hin*dIII, yielding pCMF-TC.

### Rice transformation and growth conditions

pCMF-TC was introduced into *A*. *tumefaciens* strain LBA4404 for genetic transformation of rice (*O*. *sativa* subsp. *japonica* cv. Dongjin). Rice transgenic plants were generated by the *Agrobacterium*-mediated cocultivation method [31], and transformants were selected based on hygromycin resistance. The regenerated shoots were transferred to Murashige and Skoog (MS) solid medium for root induction for 2 weeks and then transferred to 0.1% Hyponex solution (Hyponex, Imlay, MI, USA) for 1 week in a culture room for acclimatization. Regenerated plantlets were subsequently transplanted into the soil in pots and grown in a greenhouse. Transgenic rice plants and seeds were sown in a vermiculite and potting soil mixture soaked with water. All plants were grown under white fluorescent light (600 μmol m^-2^ s^-1^, 12 h photoperiod) and 75% relative humidity at 28°C in a greenhouse.

### PCR analysis

Plant genomic DNA for PCR was isolated from fresh leaves and purified using a DNeasy Plant Mini kit (Qiagen GmbH, Hilden, Germany), according to the manufacturer’s instructions. The DNA concentration was determined using a NanoDrop Spectrophotometer ND-1000 (NanoDrop Technologies, Wilmington, DE, USA) at 260 nm wavelength. A typical 25-μL PCR tube contained 10 ng genomic DNA, 200 nM of each primer, 200 μM dNTPs, 1× PCR buffer, and 1 U Taq DNA polymerase (Takara Bio, Shiga, Japan). The PCR was carried out at 94°C for 5 min, followed by 34 cycles of denaturation at 94°C for 1 min, annealing at 65°C for 1 min, and extension at 72°C for 1 min, with a final extension for 5 min at 72°C using a TProffesional TRIO thermocycler (Biometa, Berlin, Germany). Transgenic plants were analyzed with P35SF (5′-TCAGAAGACCAGAGGGCTATTGAGACTTTTCA-3′) and HPTR (5′-AATTGCCGTCAACCAAGCTCTGATAGAGTTG-3′) primers for transformation and P35SF and TCR (5′-GCAGTCAAAGCAACATCTCCAATCGC-3′) primers for gene excision.

### Southern blot analysis

Genomic DNA was isolated from mature leaves of rice using the CTAB DNA extraction method [32]. About 20 μg of total DNA was digested overnight with *Eco*RV. The digested DNA was separated on 0.8% agarose gels and transferred onto a Hybond-N+ membrane (Amersham Biosciences, Piscataway, NJ, USA). The blot was hybridized with the DIG-labeled probe, and the signal was detected with the Fusion FX imaging system (Vilber Lourmat, Eberhardzell, Germany). DNA probes for hygromycin and the tocopherol cyclase gene were obtained by PCR using the following primers: *hpt*, 5′-TTCTGATCGAAAAGTTCGACAGCGTCTCC-3′ and 5′-AATTGCCGTCAACCAAGCTCTGATAGAGTTG-3′; and *NtTC*, 5′-AATTTGAGCACTTTTCCGTCTACCTTGAAGCTG-3′ and 5′-CAGCAGTCAAAGCAACATCTCCAATCGC-3′. DNA probe preparation, hybridization, and membrane washing were performed using a DIG High Prime DNA Labeling and Detection Starter Kit II according to the manufacturer’s instructions (Roche Inc., Mannheim, Germany).

### Reverse transcription (RT)-PCR analysis

To examine transgene expression and gene excision, total RNA leaves was extracted from powdered leaf tissue using an RNeasy Mini Kit and on-column DNase treatment according to the manufacturer’s protocol (Qiagen, Valencia, CA, USA). Two micrograms of total RNA was reverse-transcribed using SuperScript II Reverse Transcriptase with oligo(dt) primers (Invitrogen, Carlsbad, CA, USA) according to the manufacturer’s instructions. PCR amplification was carried out for 28–34 cycles using 100 ng of cDNA and gene-specific primers. The primers used to amplify *hpt*, *NtTC*, and *Tub* were as follows: *hpt*, 5′-TTCTGATCGAAAAGTTCGACAGCGTCTCC-3′ and 5′-AATTGCCGTCAACCAAGCTCTGATAGAGTTG-3′; *NtTC*, 5′-CAGCAGTCAAAGTAACATCTCCAATCGC-3′ and 5′-AATTTGAGCACTTTTCCGTCTACCTTGAAGCTG-3′; and *Tub*, 5′-AAGGAGGGAGTGGGTAGAGAGGACACTGTTGTAT-3′ and 5′-TGCTTTCAACACCTTCTTCAGTGAGACTGGTG-3′. PCR was performed under the following conditions: 10 min at 94°C; 28–34 cycles at 94°C for 30 s, 65°C for 30 s, and 72°C for 1 min; and a final extension at 72°C for 10 min.

### Seed germination assay

Self-pollinated seeds were harvested from wild-type and T_0_ transgenic rice plants, surface-sterilized for 10 min in 1.5% sodium hypochloride solution, and rinsed thoroughly (3–4 times) in sterile water. Surface-sterilized seeds were grown on germination medium (MS medium 2% [w/v] sucrose and 0.7% [w/v] agar) containing hygromycin (30 mg L^-1^) or without any antibiotics. Ten days later, seedlings grown on MS media were evaluated for resistance.

### Tocopherol extraction and analysis

Mature seeds harvested from T_1_ transgenic rice plants and wild-type rice seeds were husked and ground to a fine powder using a stainless-steel planetary ball mill (Pulverisette 6, Fritsch GMBH, Germany). Alpha- and gamma-tocopherol in rice seeds were quantified by gas chromatography (GC) coupled with time-of-flight mass spectrometry (TOF-MS) as described by Park et al. [33]. Quantitative calculations were based on the corrected peak area ratios relative to the peak area of the internal standard (IS). Linearity was tested by least-squares regression analysis of the corrected peak area ratios against increasing weight ratios (relative to IS).

## Results

### Construction and characteristics of pHWMF, pCMF, and pCMF-TC vectors

**Fig 1:**
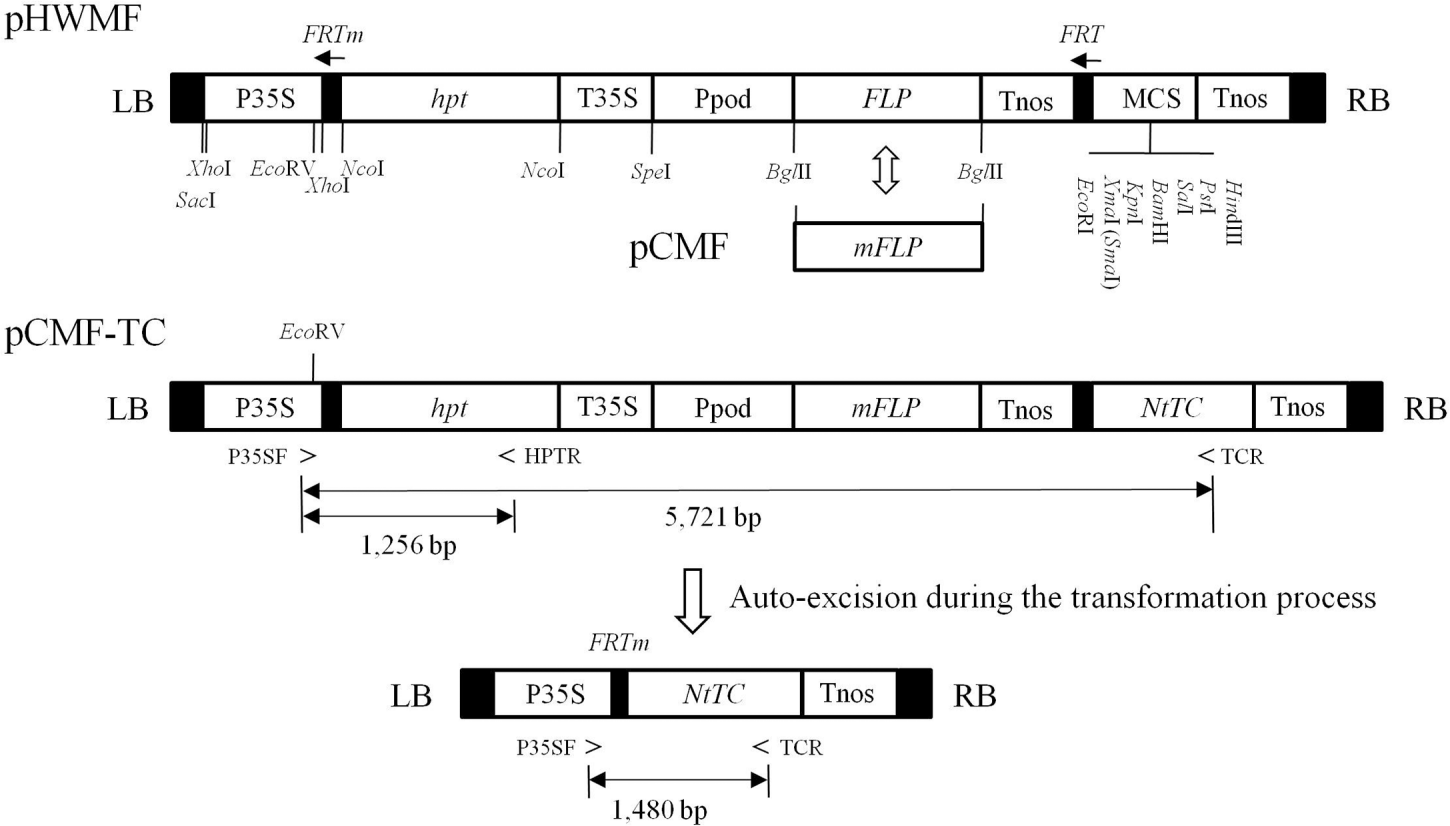
Schematic diagram of the T-DNA region of binary vectors for marker elimination and an DNA excised product. Restriction sites within the MCS were unique digestion sites in the vector. The structure of pCMF was the same to as of pHWMF except that the modified *FLP* gene was replaced with the native *FLP* gene. pCMF-TC was derived from inserting the *NtTC* gene, which is a tocopherol cyclase ortholog isolated from tobacco, into multiple cloning sites of pCMF. After gene excision, the CaMV 35S promoter was inserted adjacent to the *NtTC* coding region. P35SF, HPTR, and TCR primers were designed to detect the DNA excision. The PCR product amplified with P35SF/TCR would be 1.5 kb if DNA excision occurred and 5.7 kb otherwise. P35S, CaMV 35S gene promoter; *hpt*, hygromycin phosphotransferase gene; T35S, 35S CaMV gene terminator; Ppod, stress-inducible peroxidase gene promoter; *FLP*; recombinase gene from *Saccharomyces cerevisiae*; Tnos: *Agrobacterium* nopaline synthase gene terminator; MCS, multiple cloning site; LB, left border; RB, right border; *NtTC*, tocopherol cyclase gene isolated from tobacco; *FRT*, FLP recognition site.

The region between the left and right T-DNA borders in the binary vector pHWMF is depicted schematically in Fig 1, in which the hygromycin-selectable marker gene (*hpt*) and the recombinase gene *FLP* under the control of the oxidative stress-inducible promoter of the sweet potato were placed between two opposite *FRT* sites.

The multiple cloning sites contained unique restriction sites, where the gene of interest could be inserted, located between the *FRT* site and nopaline synthase (*nos*) gene terminator. The pHWMF vector was constructed with modular components having unique pairs of restriction sites flanking each genetic element within the T-DNA region, enabling the replacement of each of genetic element by equivalent counterparts with ease. Additionally, we used the modified *FRT* site (*FRTm*) and one full-length *FRT* site in the pHWMF vector. Although *FRTm* contained only two symmetry elements for the FLP protein compared with the full-length *FRT* sequence having three FLP binding sites, this modification did not substantially affect the function of the FLP recombinase and provided a means to clearly distinguish site-specific recombination products from possible artifacts generated by other genomic DNA modifications [34].

Genes with a high GC content are generally expressed at high levels in rice [30]. Furthermore, codon bias has a significant influence on protein yields. When we compared the codon bias of the native *FLP* recombinase gene from yeast with the codon usage in coding sequences of *O*. *sativa* using the codon usage database, we found distinct differences (Table 1).

**Table 1:** Differences in codon usage between *Oryza sativa*, native *FLP*, and *S*. *cerevisiae* and codons used for *mFLP* gene synthesis.

Amino acid	Codon	RCSU			Amino	Codon	RCSU		
		*O*. *sativa*	*FLP*	*S*. *cerevisiae*	acid		*O*. *sativa*	*FLP*	*S*. *cerevisiae*
Ala	GCA^+^	0.73	1.83	1.15	Ser	AGC*^+^	1.22	1.46	0.66
	GCC*	1.31	0.67	0.90		AGU^+^	0.67	1.02	0.95
	GCG*	1.13	0.17	0.44		UCA^+^	0.95	1.76	1.26
	GCU^+^	0.83	1.33	1.51		UCC*	1.24	0.15	0.96
Cys	UGC*	1.33	-	0.74		UCG	0.95	0.73	0.58
	UGU^+^	0.67	2.00	1.26		UCU	0.97	0.88	1.59
Asn	AAC*^+^	1.10	1.43	0.82	Tyr	UAC*^+^	1.21	1.05	0.88
	AAU	0.90	0.57	1.18		UAU	0.79	0.95	1.12
Pro	CCA^+^	0.99	1.86	1.67	Val	GUA^+^	0.41	1.05	0.84
	**CCC**	0.83	0.27	0.62		GUC*	1.21	0.85	0.84
	CCG*	1.24	0.27	0.48		GUG*^+^	1.45	1.05	0.76
	CCU^+^	0.94	1.60	1.23		GUU^+^	0.93	1.05	1.56
Lys	AAA^+^	0.66	1.11	1.15	Thr	ACA^+^	0.95	1.86	1.21
	AAG*	1.34	0.89	0.85		ACC*	1.23	0.43	0.87
Asp	GAC*	1.05	0.13	0.70		ACG	0.94	0.43	0.54
	GAU^+^	0.95	1.87	1.30		ACU^+^	0.88	1.28	1.38
Leu	CUA^+^	0.51	1.35	0.85	Arg	AGA^+^	0.90	2.10	2.89
	CUC*	1.71	0.15	0.34		AGG*^+^	1.38	2.10	1.25
	CUG*	1.39	0.60	0.66		CGA	0.55	0.60	0.40
	CUU*	1.01	0.90	0.78		CGC*	1.39	0.30	0.35
	UUA^+^	0.41	1.65	1.65		CGG*	1.16	0.30	0.24
	UUG^+^	0.97	1.35	1.72		CGU	0.62	0.60	0.87
His	CAC*	1.10	0.89	0.73	Gly	GGA^+^	0.82	2.00	0.86
	CAU^+^	0.90	1.11	1.27		GGC*	1.53	0.75	0.77
Phe	UUC*	1.26	0.80	0.83		GGG	0.88	0.25	0.48
	UUU^+^	0.74	1.20	1.17		GGU	0.77	1.00	1.89
Gln	CAA	0.79	0.94	1.39	Ile	AUA^+^	0.62	1.42	0.82
	CAG*^+^	1.21	1.06	0.61		AUC*	1.38	0.75	0.79
Glu	GAA^+^	0.72	1.15	1.41		AUU	1.00	0.83	1.39
	GAG*	1.28	0.85	0.59					

An asterisk (*) after the codon indicates a favored codon (RCSU > 1) in genes of coding DNA sequences in the rice genome, and a plus sign (+) indicates a favored codon (RCSU > 1) in the native *FLP* gene. Codons used for synthesis of the *mFLP* gene are shown as bold text and were favored codons in high-GC genes within the rice genome [30]. Relative synonymous codon usage (RSCU) was defined as the ratio of the observed frequency of codons to the expected frequency given that all synonymous codons for the same amino acids were used equally.

For example, proline can be encoded by the codons CCU, CCA, CCC, and CCG. However, the amino acid proline in the native *FLP* gene was mostly encoded by the triplets CCU and CCA, while CCC and CCG were rare codons. In contrast, CCG was the favored codon for proline in *O*. *sativa* and was used significantly more often in high-GC genes than in low-GC genes (GC content < 60%) in the rice genome. Furthermore, the *S*. *cerevisiae FLP* gene had a GC content of 37%. In contrast, the nuclear genome of *O*. *sativa* has an overall GC content of 43% [35], with coding regions having a GC content of 55% (as determined using the codon usage database). Due to these differences between *S*. *cerevisiae* and *O*. *sativa*, we synthesized the modified *FLP* recombinase (*mFLP*) gene with a bias for preference in high-GC genes of rice. The resulting sequence had a GC content of 61% with 70% DNA homology to the native *FRT* recombinase gene (S1 Fig). The *mFLP* gene was substituted with the native *FLP* gene of the pHWMF vector to generate the pCMF binary vector.

Tocopherol cyclase (TC/VTE1) catalyzes the conversion of 2,3-dimethyl-5-phytyl-1,4-benzoquinone (DMPBQ) to γ-tocopherol (S2 Fig). Previous studies have reported the characterization of *NtTC*, a tocopherol cyclase ortholog originating from tobacco, and have demonstrated a relative increase in tocopherol content in the leaves of transgenic rice plants upon overexpression of *NtTC* [27]. To enhance the tocopherol contents of rice seed, the plasmid binary pCMF-TC was constructed as shown in Fig 1 and introduced into *A*. *tumefaciens* strain LBA4404 for rice transformation.

### Rice transformation and identification of transgenes in T_0_ rice plants

**Fig 2:**
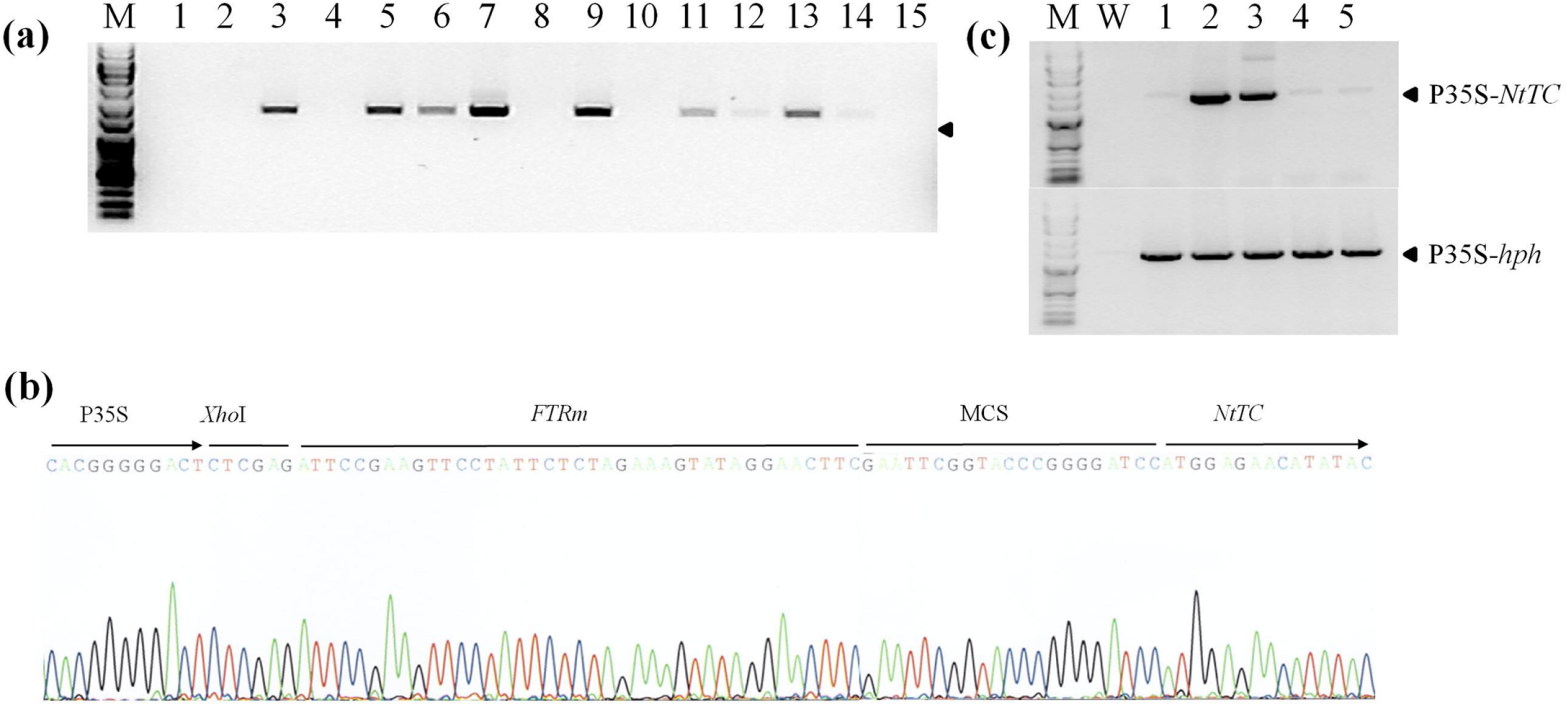
PCR and sequencing analysis for identification of transgenes and gene excision in transgenic T_0_ plants. (a) PCR results with the primer pair P35S/TCR using the genomic DNAs prepared from fresh leaves of transgenic plants after regeneration. (b) Sequence analysis to confirm gene excision from the genome of transgenic T_**0**_ plants. The PCR fragments amplified using the primers P35S and TCR were sequenced. (c) PCR results with the primer sets P35SF/HTPR and P35SF/TCR using genomic DNAs prepared from the mature leaves of five randomly selected transgenic T_**0**_ lines. M, molecular marker. Line numbers are indicated at the top of each lane.

A total of 14 regenerated rice plants were obtained after *Agrobacterium*-mediated transformation and hygromycin selection. Gene integration and excision from T-DNA-regenerated rice plants was first verified by PCR with genomic DNA extraction from fresh leaves using P35SF and TCR primers specific for the CaMV 35S promoter and *NtTC* gene, respectively. The results, shown in Fig 2A, revealed the presence of a PCR band of about 1.5 kb in many regenerated plants; this was consistent with the expected size of the PCR amplicon for the excised T-DNA with the P35S-*NtTC* fragment. PCR amplification of the full T-DNA without excision did not yield a visible band presumably due to the large size of the amplicon (5,721 bp). To further validate that DNA excision had occurred in the *FRT* site, the PCR fragments were sequenced, as shown in Fig 2B. The sequencing result showed correct sequences matching the *FRTm* site and flanked *NtTC* sequences, as expected from the recombinant T-DNA.

The regenerated rice plants were planted in a greenhouse, and five lines were then randomly chosen to reconfirm the excision of T-DNA for further analysis. The presence of the integrated T-DNA into the rice genome was reassessed by PCR with the gene specific primer sets P35SF/HTPR and P35SF/TCR. PCR results indicated that the 1,256-bp P35S-*hpt* fragment could be detected in all of five plant lines, while the 1,480-bp P35S-*NtTC* fragment was detectable in only two plant lines, indicating that these transgenic lines contained multiple T-DNAs with excised and unexcised sequences. These cotransformed plants were designated TC2 and TC3.

### Selection of marker-free transgenic rice plants (T_1_) expressing a *NtTC* gene

**Fig 3:**
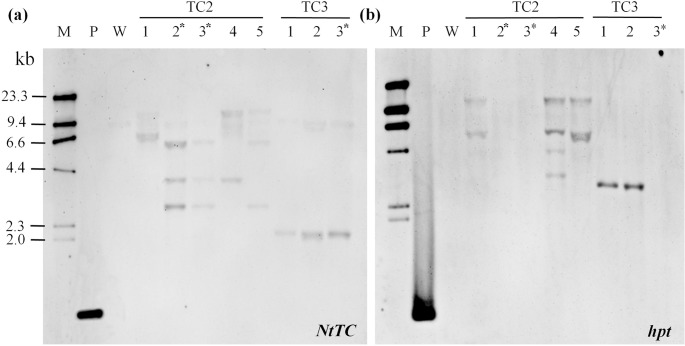
Southern blot analysis of T_1_ progenies of the transgenic lines. Genomic DNA was digested with *Eco*RV, which created one cut in pCMF-TC, and hybridized with the DIG-labeled *NtTC* (a) or *hpt* (b) probe. M, DIG-labeled molecular marker; P, positive control; lanes 1–5, progenies of the TC2 line; lanes 6–8, progenies of the TC3 line.

If a transgenic plant harbored the excised T-DNA and unexcised full T-DNA in unlinked loci, genetic separation of excised marker-less T-DNA from the selectable marker gene may be feasible by segregation in the T_1_ generation. Therefore, some of T_1_ progenies from the TC2 and TC3 line were analyzed for the presence of the *hph* and *NtTC* transgene by Southern blotting. All five T_1_ progenies of the TC2 line and three T_1_ progenies of the TC3 line were randomly chosen, and nontransformed wild-type Dongjin was used as control for Southern blotting. All of these T_1_ plants exhibited multiple *NtTC* bands, and progenies of crosses between the TC2 and TC3 lines showed different band patterns representing independent transgenic lines. In addition, a weak 9.4-kb band also appeared in the wild-type strain; this band may have been associated with the endogenous rice tocopherol cyclase gene, which exhibited high similarity with the *NtTC* probe. In the case of hybridization with the *hpt* probe, two T_1_ progeny of the TC2 line (TC2-2 and TC2-3) and one T_1_ progeny of the TC3 line (TC3-3) did not exhibit a signal, thus confirming complete excision (Fig 3).

**Fig 4:**
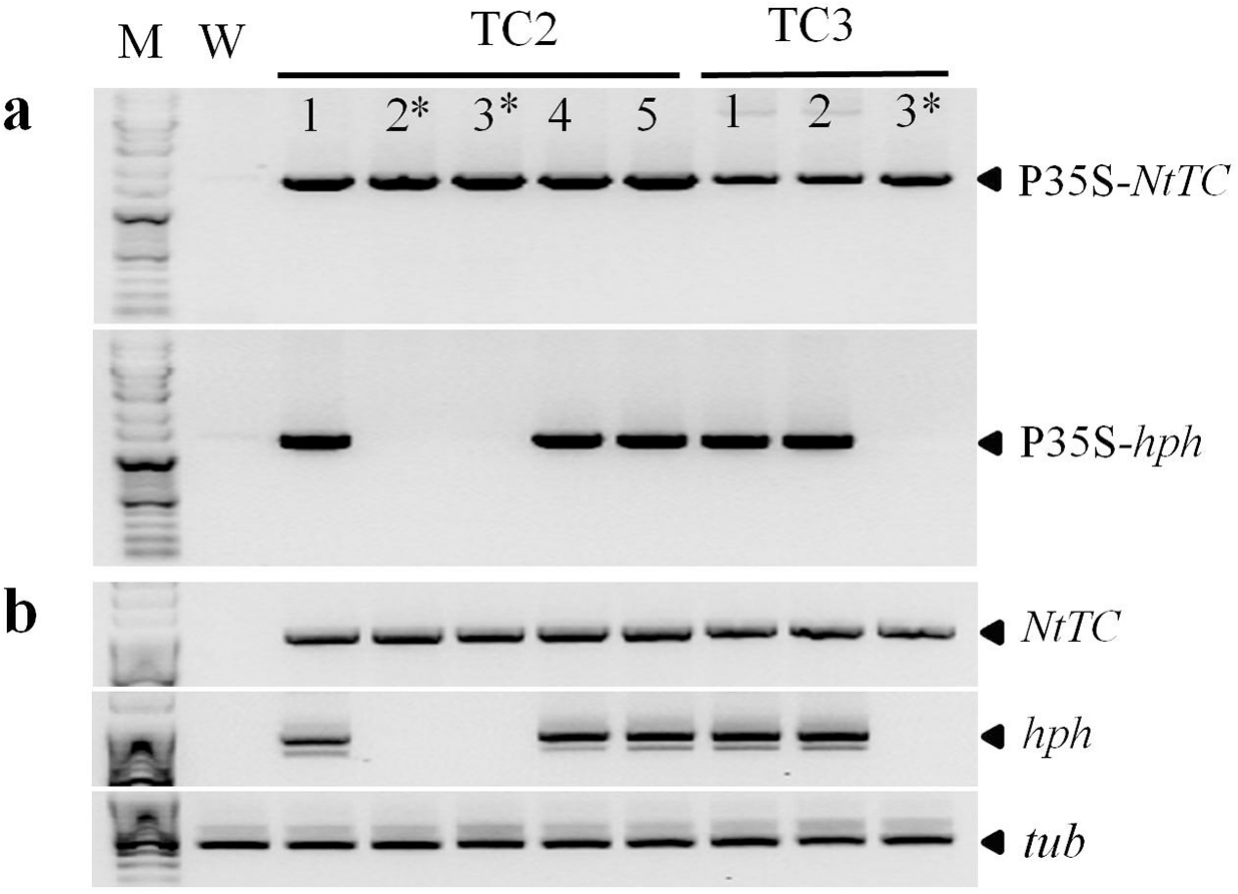
Identification of the T-DNA in transgenic rice plants to select marker-free transgenic rice lines. (a) PCR analysis of T_**1**_ transgenic rice plants for marker excision. (b) RT-PCR analysis of *NtTC* and *hpt* from leaves of T_**1**_ transgenic rice plants. The rice tubulin (*tub*) gene was used for normalization.

To validate these Southern blotting results, the excision status of T_1_ rice progeny from transgenic lines TC2 and TC3 was also analyzed by PCR using the P35SF/HTPR and P35SF/TCR primer sets (Fig 4A). PCR analyses indicated the presence of the expected P35S-*NtTC* fragment and the absence of the P35S-*hpt* gene in the TC2-2, TC2-3, and TC3-3 lines, consistent with Southern blot analysis data. Moreover, these marker-free rice lines did not exhibit expression of the *hpt* gene but expressed high levels of *NtTC*, as shown by RT-PCR analysis (Fig 4B).

**Fig 5:**
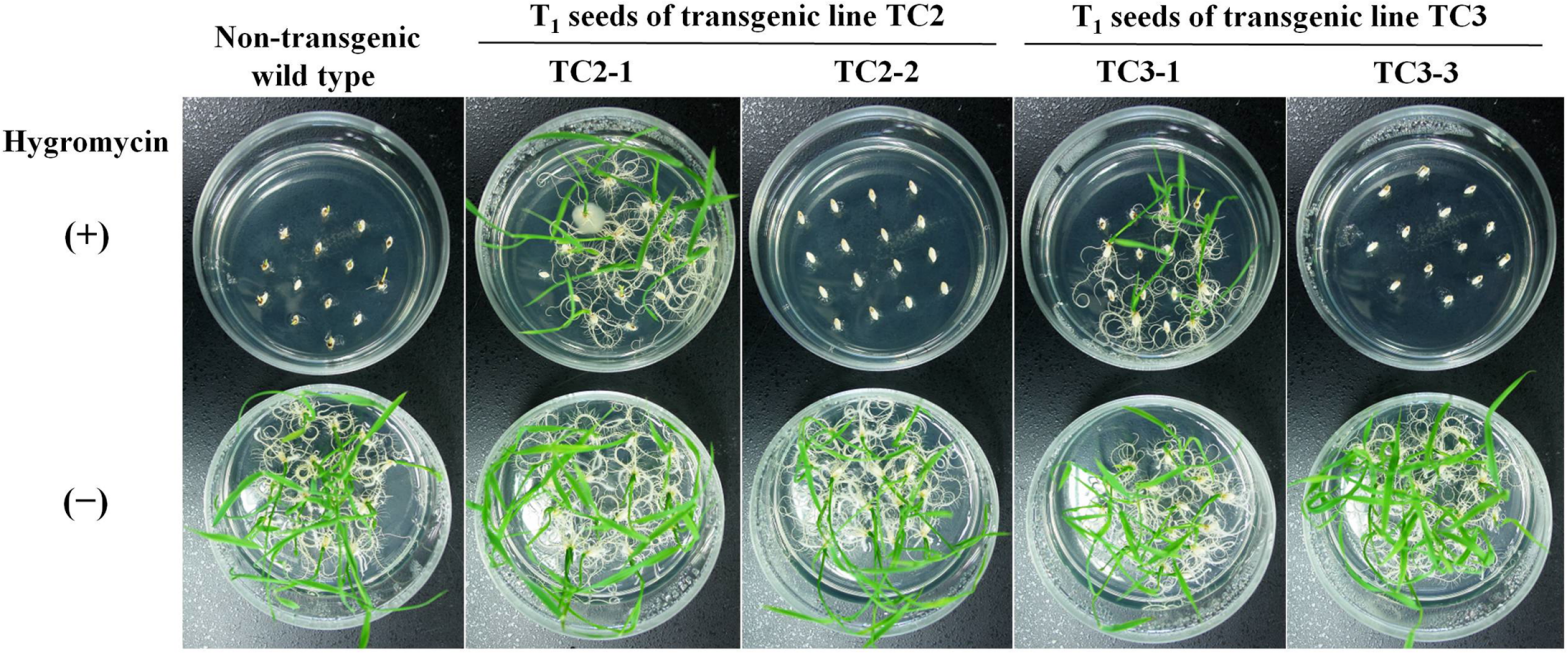
Germination test of T_1_ progenies derived from the transgenic lines. Seeds of the transgenic primary plants and nontransgenic wild-type plants were sown on germination medium containing hygromycin or without antibiotics.

Next, we determined the hygromycin resistance of T_1_ seeds of transgenic rice lines. The four transgenic lines confirmed above, in which the *hpt* gene was excised or existed, were chosen for resistance analysis. Rice seeds derived from the TC2 and TC3 lines that had been selfed were sown on hygromycin-supplemented medium. As shown in Fig 5, the TC2-1 and TC3-1 lines, which were chimeric with excised and unexcised T-DNA loci coexisting in the chromosomal DNA, grew on hygromycin-supplemented medium, while TC2-2 and TC3-3 did not, indicating that the *hpt* gene was excised successfully by mFLP/*FRT*-mediated marker gene excision. In addition, transgenic plants grew normally and showed no visually detectable phenotypic differences from nontransgenic wild-type plants in the field and greenhouse.

### Tocopherol contents of transgenic rice grains

The TC3-1 transgenic rice line, carrying a single copy of the transgene in its genome, and the untransformed controls were grown in the same field under normal culture conditions, and mature seeds were harvested for determination of tocopherol content using GC coupled with TOF-MS. In rice, β- and δ-tocopherol usually appear in trace amounts, while α-tocopherol and γ-tocotrienol are the most abundant isomers [36,37]. Therefore, these two isomers were not included, and the total tocopherol content is given here as the sum of α- and γ-tocopherol.

**Fig 6:**
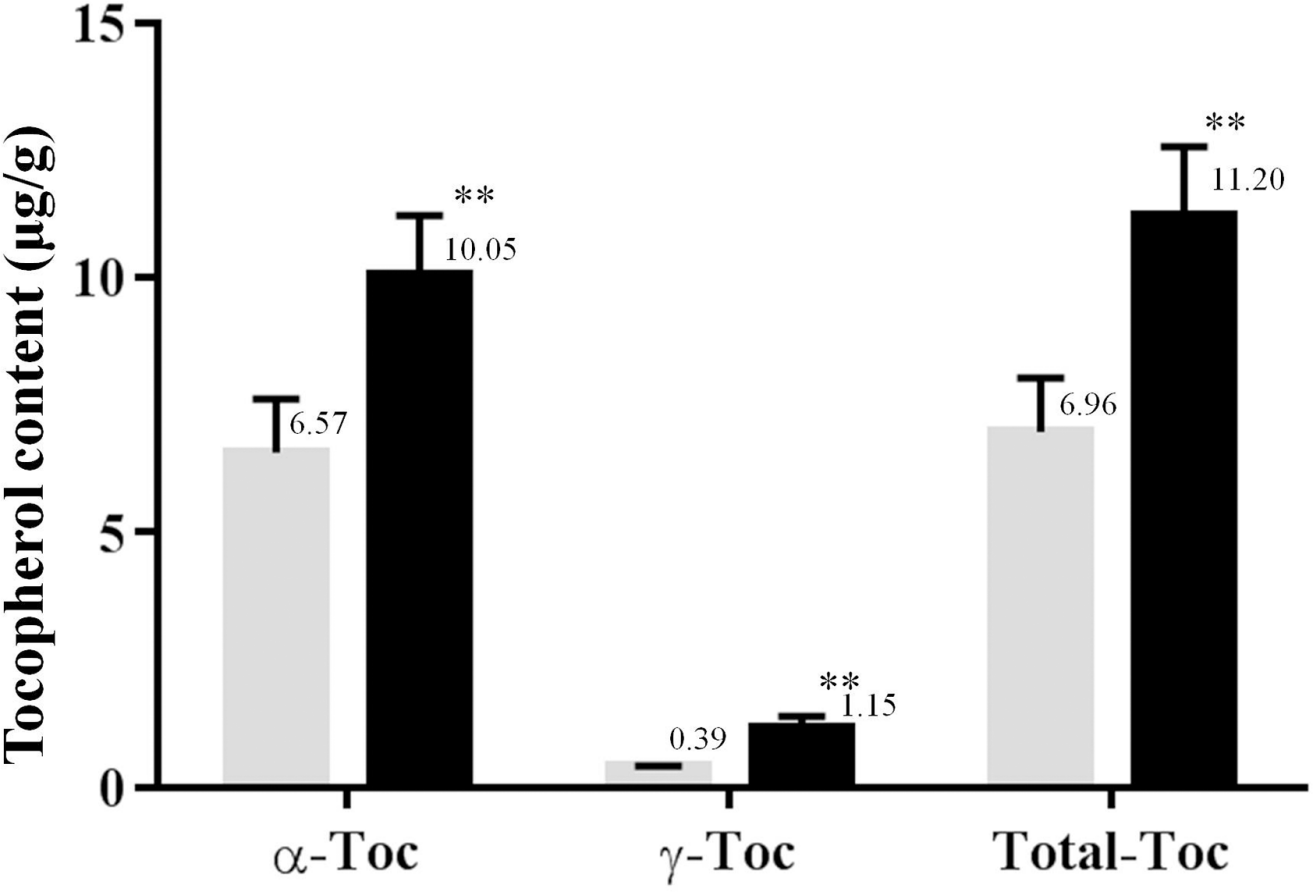
Tocopherol contents from seeds of marker-free transgenic rice plants and wild-type rice plants. Black and gray bars correspond to transgenic TC3-1 plants and wild-type rice plants. Values are means ± SDs (n = 5), and student’s t tests were used to compare values obtained from transgenic lines to those of the wild-type plants. ** *p* < 0.01.

The contents of α-, γ-, and total tocopherol in the seeds of wild-type rice plants were 6.57 ± 1.05, 0.39 ± 0.03, and 6.96 ± 1.06 μg/g dry weight, respectively. In the case of the transgenic TC3-1 line, α-, γ-, and total tocopherol levels were approximately 53%, 194%, and 61% higher than those in seeds of wild-type plants (Fig 6; *p* < 0.01). These results demonstrated that overexpression of *NtTC* could increase tocopherol levels in rice seeds.

## Discussion

Selectable marker genes, such as antibiotic- or herbicide-resistant gene, are powerful selection tools for use in plant transformation processes. However, once transformation is accomplished, the presence of these resistance genes is no longer necessary and can even be undesirable. Therefore, various genetic approaches have been developed to produce marker-free transgenic plants, and several techniques, including cotransformation and auto-excision using site-specific recombination, have been successfully established for the elimination of selectable marker genes [11, 14, 38]. However, these methods still require additional steps or more labor to obtain marker-free transgenic plants, and further improvements are needed for practical application and conventional use. Accordingly, in this study, we developed a spontaneous self-excision binary vector using an oxidative stress-inducible modified FLP/*FRT* system. This system was successfully applied to produce marker-free transgenic rice plants with enhanced seed tocopherol content.

The oxidative-stress inducible POD promoter from sweet potato was not expressed in any tissues in differentiated plants, but was strongly expressed and induced by environmental stress conditions, including hydrogen peroxide and wounding in cultured cells [39]. Also, spontaneous excision of a marker gene in an oxidative stress-inducible FLP/*FRT* system was reported in tobacco plants without chemical induction, presumably resulting from activity of the POD promoter in transgenic cells during the transformation process [39].

In this study, in order to develop a simple and convenient method of producing marker-free transgenic rice plants using the oxidative-stress inducible FLP/*FRT* system, we synthesized the pHWMF vector, in which the site-specific recombinase gene *FLP* and selectable marker gene *hpt* were inserted between two *FRT* sites. The *FLP* gene was under the control of oxidative-stress inducible POD promoter; thus, both the selectable marker and *mFLP* genes could be excised spontaneously in the rice callus stage of transformation. However, when the pHWMF vector was introduced into *E*. *coli* or *A*. *tumefaciens*, weak gene excision was observed in our experiments, possibly due to the expression of the *FLP* gene driven by leakiness of POD promoter activity. This phenomenon has been reported by other laboratories using chemical- or heat shock-inducible promoters for recombinase gene expression [40] and could be associated with the maintenance of plasmid constructs and transformation efficiency. To solve this problem, the *FLP* gene was modified based on the codon usage criteria for rice and was altered to have elevated GC content. As a result, pCMF containing the *mFLP* gene controlled by the POD promoter was stably maintained in *E*. *coli* and *Agrobacterium* (data not shown). Additionally, this modification may contribute to the efficient expression of the construct in rice and other cereals with the same GC content preference.

**Fig 7:**
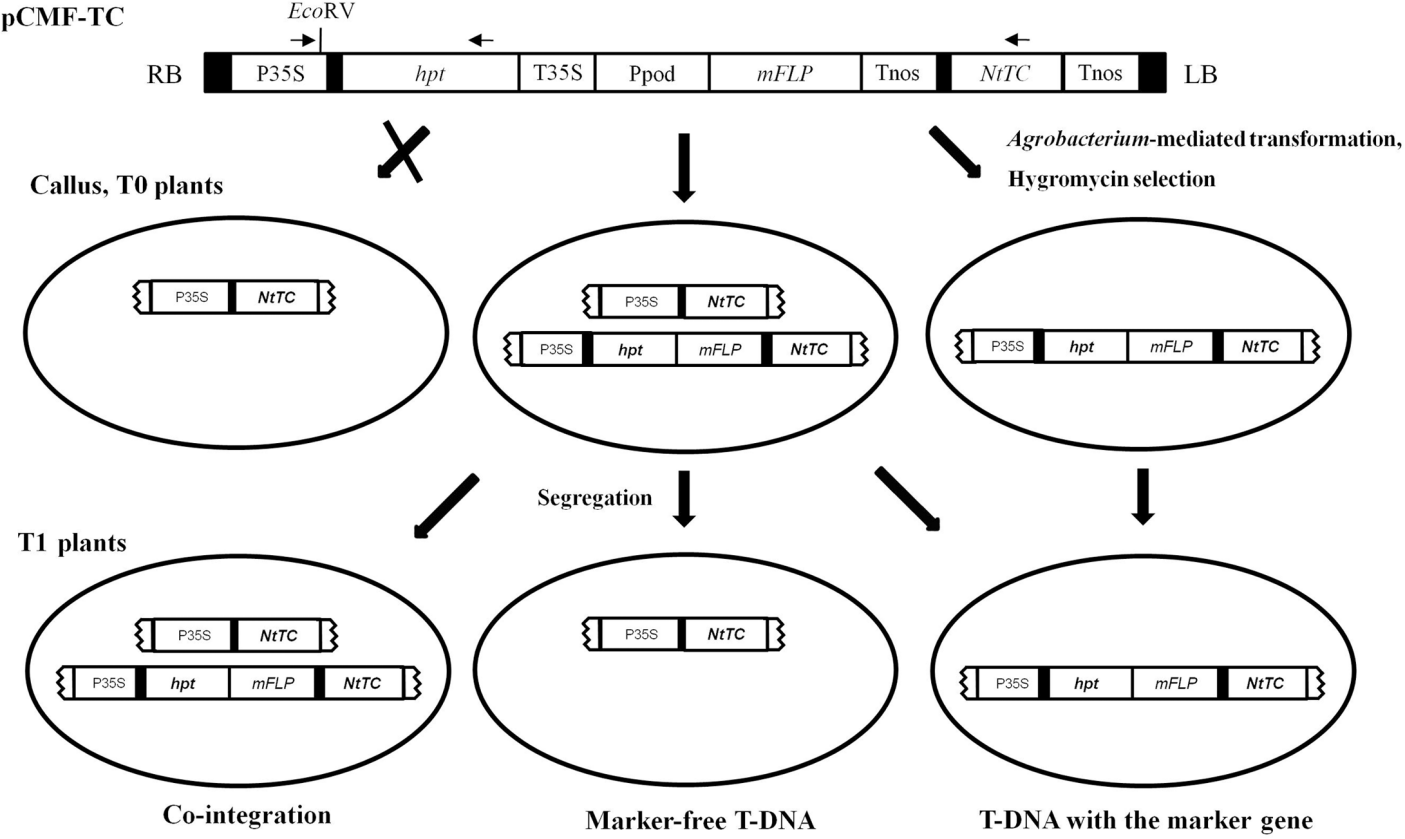
Schematic showing the production of selectable marker-free transgenic rice.

In *Agrobacterium*-mediated transformants, transformed T-DNAs are often integrated into different loci in the plant genome, and more than one-third contain a single T-DNA insert, while half contain 2–3 copies and the remainder (about 15%) contain 4–5 copies [41]. The principle of the spontaneous auto-excision strategy doe generation of marker-free transgenic rice plants is the introduction of multiple T-DNAs. If the T-DNAs were integrated into unlinked loci, and gene excision occurred in some of the T-DNAs resulting from stress stimuli during the transformation procedure, excised T-DNA was inherited independently, consequently producing marker-free transgenic plants in the subsequent T_1_ generation (Fig 7). Although we did not attempt to analyze the excision rates of all regenerated transgenic plants, about half of regenerated plants obtained by hygromycin selection presented a P35S-*NtTC* fragment, representing the expected PCR amplification band of an excised T-DNA. Moreover, marker-free T_1_ transgenic rice plants were generated from two T_0_ transgenic lines among the five transgenic lines analyzed in this study. Thus, this method was effective for production of marker-free transgenic rice plants. Compared with other methods for developing marker-free transgenic plants, this spontaneous auto-excision strategy has various unique advantages. For example, this strategy does not require additional procedures for eliminating the selectable marker gene, such as crossing of the recombinase gene or chemical treatment, and can therefore be applied with general rice transformation methods to obtain marker-free rice plants.

We attempted to produce the marker-free transgenic rice plants overexpressing *NtTC* using the pCMF-TC vector in order to verify efficient generation of marker-free transgenic rice plants via the spontaneous auto-excision method and improve the tocopherol content of rice seeds. Tocopherols, which are collectively known as vitamin E, are essential antioxidant components of both human and animal diets, and the main source for the dietary uptake of tocopherols is plants, consumed as seeds, vegetables, fruits, and plant seed oils. Hence, increased levels of tocopherols in rice grains would be greatly beneficial for human health. Here, we demonstrated that overexpression of *NtTC* under the CaMV 35s promoter resulted in increases levels of total tocopherols by approximately 1.6-fold in T_2_ seeds of marker-free transgenic rice plants. In addition, γ-tocopherol content was significantly increased by 194% compared to that in wild-type plants. Similar results have also been reported in other transgenic rapeseed plants expressing the *TC* gene from *Arabidopsis* or maize; indeed, the seeds of transgenic *B*. *napus* plants exhibit significantly enhanced tocopherol content by a factor of 1.6 [42]. Thus, these results implied that TC activity may be the limiting factor for tocopherol biosynthesis, and overexpression of *TC* influences the total tocopherol content of plants.

In conclusion, the results reported herein demonstrate that the spontaneous auto-excision method using a FLP/*FRT* recombination system is able to produce marker-free transgenic rice plants with enhanced seed tocopherol content by *Agrobacterium*-mediated transformation with antibiotic selection. This method is simple and does not require additional procedures to eliminate the selectable marker gene. Hence, this method provides a useful tool for production of marker-free transgenic rice plants. Importantly, this system may also be applicable to other cereals. Additionally, the nutritional enhancement of rice seeds through elevation of tocopherol content coupled with this marker-free strategy may improve human health and public acceptance of GM rice.

## Supporting Information

S1 Fig DNA sequence alignment of the native and modified *FLP* genes. The alignment was created using ClustalW2 software with sequences available in the EMBL database. DNA sequences conserved within the alignment are designated with an asterisk (*).
(TIF)

S2 Fig Simplified tocopherol biosynthetic pathway. HGA, homogentisic acid; PDP, phytyl-diphosphate; DMPBQ, 2,3-dimethyl-5-phytyl-1,4-benzoquinone; MPBQ, 2-methyl-6-phytyl-1,4-benzoquinone; HPT/VTE2, homogentisate phytyltransferase; TC/VTE1, tocopherol cyclase; MPBQ MT/VTE3, MPBQ methyltransferase; γ-TMT/VTE4; γ-tocopherol methyltransferase.
(TIF)
