# Supplementary figures and images for: Development of Selectable Marker-Free Transgenic Rice Plants with Enhanced Seed Tocopherol Content through FLP/FRT-Mediated Spontaneous Auto-Excision
Source: PLoS One. 2015 Jul 14;10(7):e0132667. doi: 10.1371/journal.pone.0132667 (PMC4501831; doi:10.1371/journal.pone.0132667)

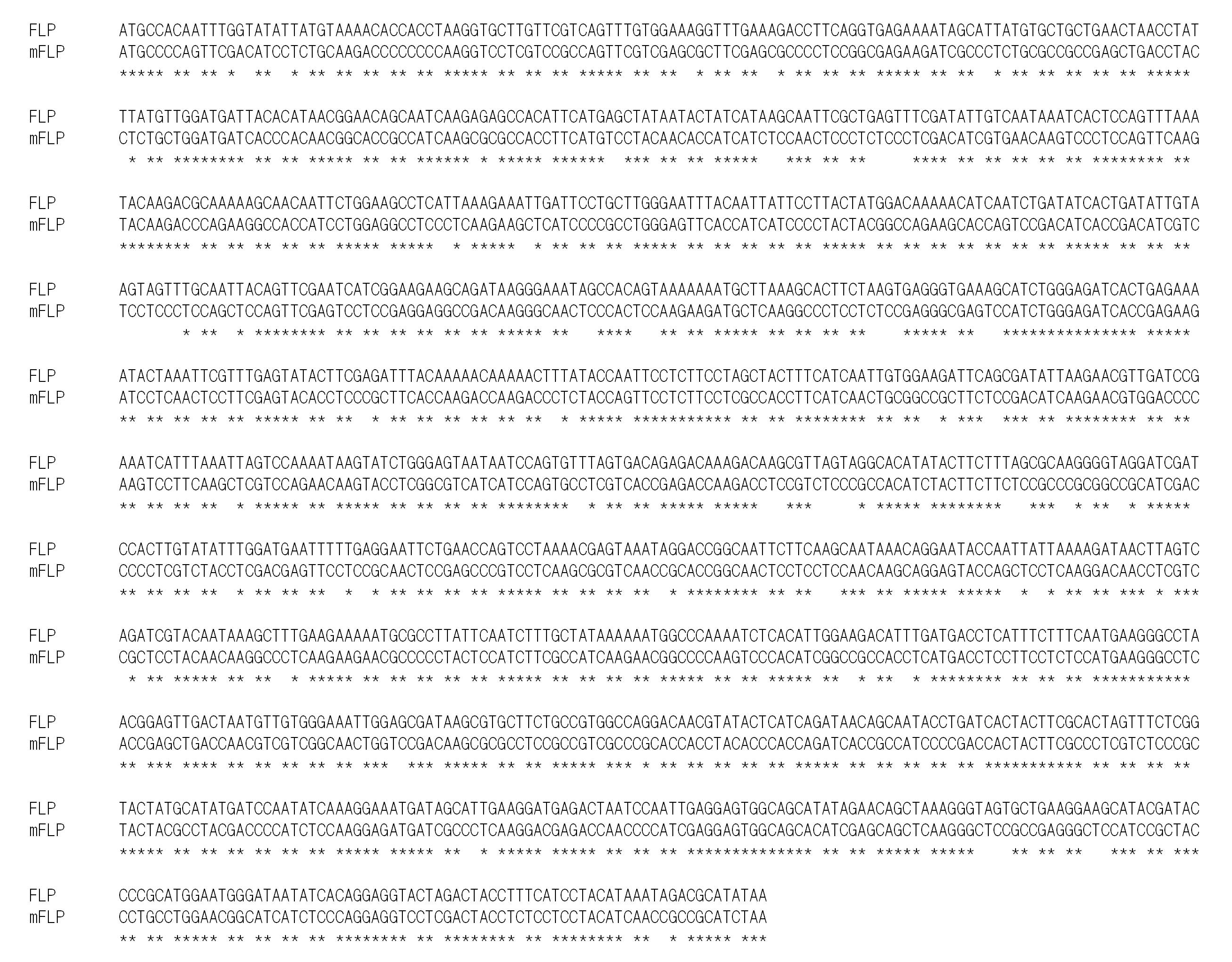

Supplement: S1 Fig — The alignment was created using ClustalW2 software with sequences available in the EMBL database. DNA sequences conserved within the alignment are designated with an asterisk (*). (TIF) [file pone.0132667.s001.tif]

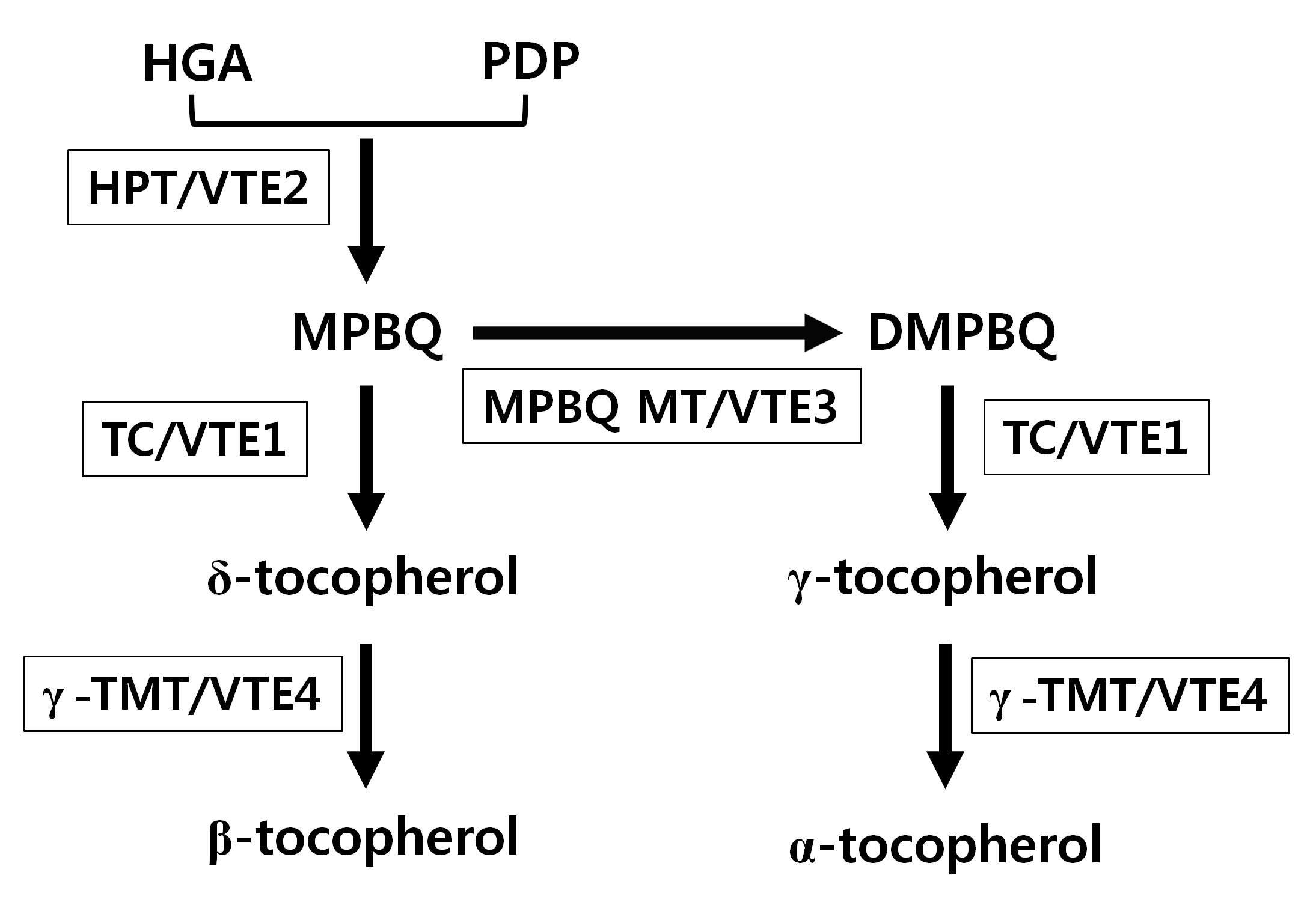

Supplement: S2 Fig — HGA, homogentisic acid; PDP, phytyl-diphosphate; DMPBQ, 2,3-dimethyl-5-phytyl-1,4-benzoquinone; MPBQ, 2-methyl-6-phytyl-1,4-benzoquinone; HPT/VTE2, homogentisate phytyltransferase; TC/VTE1, tocopherol cyclase; MPBQ MT/VTE3, MPBQ methyltransferase; γ-TMT/VTE4; γ-tocopherol methyltransferase. (TIF) [file pone.0132667.s002.tif]
